# Dynamic exergy analysis: From industrial data to exergy flows

**DOI:** 10.1111/jiec.13168

**Published:** 2021-07-19

**Authors:** Charalampos Michalakakis, Jonathan M. Cullen

**Affiliations:** Department of Engineering, University of Cambridge, Trumpington Street, Cambridge, UK

**Keywords:** energy efficiency, exergy analysis, industrial data, industrial ecology, materials efficiency, Sankey diagrams

## Abstract

As the power and transport sectors decarbonize, industrial emissions will become the main focus of decarbonization efforts. Exergy analysis provides a combined material and energy efficiency approach to assess industrial plants, both of which are necessary to tackle industrial emissions. Existing studies typically use simulated, static data that cannot inform real plant operators. This paper performs an exergy analysis on data spanning 2 years from 311 sensors of a real ammonia production site. We develop methods to overcome unique data challenges associated with real industrial data processing, visualize resource flows in Sankey diagrams, and estimate exergy indicators for both the steam methane reforming plant and its constituent processes. We evaluate average conventional and transit exergy efficiencies for the plant (71%, 15%), primary reformer (86%, 40%), secondary reformer (96%, 71%), high-temperature shift (99.7%, 77%), combustor (56%, 55%), and heat exchange section (85%, 82%). Overall exergy losses are 80 MW; the primary reformer and combustor are the two processes with the highest losses at 35 and 33 MW, respectively. Such an analysis can inform both improvement projects and performance finetuning of a real plant while being applicable to any industrial site. Increased availability of cheap wireless sensors and a shift to Industry 4.0 can enable higher resolution and real-time performance monitoring.

**Supplementary Information:**

The online version of this article (doi:10.1111/jiec.13168) contains supplementary material, which is available to authorized users.

## INDUSTRY, EXERGY, AND INDUSTRIAL DATA

### Industrial emissions and efficiency

Globally, industry was responsible for approximately a third of greenhouse gas (GHG) emissions in 2010 (Bajzelj et al., 2013), and 40% of CO_2_ emissions in 2017 (IEA, 2019), highlighting its enduring contribution to climate change. As the power, heat, and transport sectors decarbonize in the future, harder-to-decarbonize sectors such as industry will make up the majority of GHG emissions (CCC, 2019a). Additional emissions reductions, necessary to move from a 2°C warming scenario to a 1.5°C path (Luderer et al., 2018), are required in the industrial sector and demand-side interventions that improve efficiency can potentially deliver higher, more cost-effective cuts than supply-side options (Levi & Cullen, 2018; IEA, 2008).

Material and energy resources interact via multiple pathways in industrial processes with raw materials carrying “embodied” energy, that is, energy directly or indirectly used in the production of those materials; as a result both types of resources have associated “embodied” CO_2_ emissions (Davis & Caldeira, 2010; Costanza, 1980). Additionally, industrial emissions arise through various mechanisms: fuel combustion, process emissions, and non-energy use of fossil fuels (IPCC, 2018). The traditional energy efficiency approach can only deal with the former mechanism and not the others (CCC, 2019b). The consequence is that improving the resource efficiency and environmental impact of industrial plants requires a combined material and energy approach (Hernandez et al., 2018).

The field of industrial ecology offers a guiding philosophy for tackling industrial environmental impacts by seeking to analyze the relationship between industry and the environment (Ayres & Ayres, 2002). Industrial activity is placed within an ecosystem that acts as a resource provider and emission/waste sink, with the aim of minimizing waste generation and optimizing energy and material use (Suh, 2009). Exergy analysis is an important tool within industrial ecology.

### Exergy as an industrial analysis tool

Exergy is a thermodynamic quantity that can unify energy and materials into a combined approach with a single resource metric (Szargut, 2005); it can be defined as the maximum amount of work obtainable from a resource (Rant, 1956). In addition, unlike energy and materials, exergy is not conserved and can be destroyed in non-reversible processes such as chemical reactions or heat transfer (Brodyansky et al., 1994). This means that the quality of a resource, that is, its ability to provide useful work, is taken into account with exergy and thus, an exergy analysis can identify the true location and magnitude of inefficiencies in the industry (Bejan et al., 1996; Szargut et al., 1988). Several authors have argued that exergy analyses provide additional insights over energy and material metrics (Cornelissen, 1997; Sciubba, 2004; Yi et al., 2004): it takes the second law of thermodynamics into account, combines materials and energy into a single metric in a scientifically rigorous way and can identify not only external losses but also internal exergy destruction (i.e., quality loss).

Originally applied to study heat and power plants (Sciubba & Wall, 2007), exergy analysis has been widely used in numerous sectors and a large range of scales. Studies have investigated metallurgical (Morris & Steward, 1984; Szargut et al., 1988), thermal (Lazzaretto & Tsatsaronis, 2006; Kotas, 1980), chemical (Simpson & Lutz, 2007; Rivero et al., 2004), combined heat and power (CHP) (Schaumann, 2007), and oil-producing sites (Nguyen et al., 2014). Similarly, it is a versatile approach that can accommodate any scale of system being studied. Analyses have been performed at the equipment (Rakopoulos & Giakoumis, 2006), plant (Zvolinschi et al., 2007; Jokandan et al., 2015), national (Eisenmenger et al., 2017; Ayres et al., 2003), and international scale (Calvo et al., 2018; Gonzalez Hernandez et al., 2018), to name a few examples.

### Industrial data and exergy

Industrial sites currently generate significant amounts of data from the multitude of control sensors installed around the sites. With the onset of Industry 4.0, a push toward digitization of industry with the aim of, among other things, optimizing production, and enabling real-time capability (Lu, 2017), this wealth of data is only expected to increase. Coupled with improved big data analytics tools that can handle the volume generated, a vast amount of data at increasingly high resolutions can be accessed and processed. Real industrial sites operate dynamically, meaning their operating conditions fluctuate over time, as opposed to statically, where conditions stay constant. Thus, they will not continuously operate at their best performance. Analyzing dynamic industrial data can reveal how often, and how far, sites deviate from their peak performance. However, to date, typical exergy studies are performed on models or simulations, focusing on informing new process design rather than improving existing sites (Dassisti & Semeraro, 2016).

When real industrial data feed into exergy analysis such as in power plants (Açikkalp et al., 2014; Boyaghchi & Molaie, 2015; Ozdil et al., 2015) and chemical sites (Vučković et al., 2014; Ren et al., 2006), it often is used as a more accurate estimation of key operating variables in a static analysis. When dynamic data is used in some studies (Voldsund et al., 2013; Barati et al., 2017), it is often to calculate average values for these variables with the analysis still being static.

Conversely, dynamic studies rarely utilize real data and rather rely on dynamic simulations. Timeframes can be limited to single batches or days (Hatami et al., 2020; Toghyani & Rahimi, 2015). Another group of studies focus on short-term effects on exergetic indicators of step-wise disturbances in parameters such as feed temperature and flow (Montelongo-Luna et al., 2011; Jin et al., 2018; Munir et al., 2013). These studies tend to have a short timeframe and focus on control rather than resource performance.

Often, dynamic data from industrial sources suffer from missing data and metadata, outliers, noise, and time misalignment (Zhao et al., 2018; Gudivada et al., 2017). While sophisticated statistical methods exist to deal with a multitude of these issues, these methods need to be complemented with process knowledge to identify the drivers behind data corruption and contamination. In addition, they need to be computationally efficient, especially for large datasets, and preserve the underlying nature of the data such as distribution and frequency (Xu et al., 2015).

### Research gaps and objectives

Exergy analysis is a proven and known tool for assessing industrial performance and identifying both material and energy efficiency opportunities in industry. However, to the best of the authors’ knowledge, no studies have applied this tool on real industrial control data over a sufficiently long time frame to assess the fluctuations in exergy efficiency, rather than just averages. This is despite the potential to address an audience of operators and engineers at the plant level who can influence a plant’s performance.

To attempt to fill this gap, this study performs an exergy analysis on a steam methane reforming (SMR) plant producing syngas as part of an ammonia production site. The data covers 2017 and 2018, at minute-level frequency and includes 311 metering equipment. We address challenges around missing and corrupted data. The analysis is performed at two different scales: at the scale of the SMR plant and at the process scale where separate the plant into individual processes, evaluating exergy indicators. Exergy is a proxy for how useful material and energy resources are, so the terms “exergy” and “resource” are used interchangeably.

Section 2 that follows outlines the methodology used to convert industrial data to an exergy analysis. Section 3 presents the results for the plant and process-scale analyses. The results and conclusions from this study are discussed in Section 4.

## CONVERTING RAW INDUSTRIAL DATA TO AN EXERGY ANALYSIS

This section outlines the steps followed to convert raw industrial data to a dynamic exergy analysis: boundary and scale determination, modeling assumptions, and exergy methodology.

### Exergy calculations

Material flows carry kinetic, potential, physical, and chemical exergy, arising from the material’s velocity, relative position, temperature and pressure, and chemical composition, respectively (Szargut et al., 1988). Studies on industrial systems typically neglect the kinetic and potential components of exergy (Nguyen et al., 2014). The total material exergy is the sum of physical and chemical exergy, as calculated by Equations (1) and (2), respectively.

1 $$ {\dot{E}_{{\rm{ph}}}} = \dot{F}\ \ {e_{{\rm{ph}}}} = \dot{F}\ \left[ {{h_{T,P}} - {h_{{T_0},{P_0}}} - {T_0}\left( {{s_{T,P}} - {s_{{T_0},{P_0}}}} \right)} \right]. $$

Where ${\dot{E}_{{\rm{ph}}}}$ is the physical exergy flow, $\dot{F}$ is the molar flow rate, ${e_{{\rm{ph}}}}$ the specific molar physical exergy, ${h_{T,P}}$ and ${s_{T,P}}$ are the specific molar enthalpy and entropy, respectively, and ${h_{{T_0},{P_0}}}$ and ${s_{{T_0},{P_0}}}$ the specific molar enthalpy and entropy at standard conditions of 298.15 K and 1.01325 bar. All enthalpy and entropy values are calculated at their corresponding conditions using the Cantera thermodynamic library for Python (Goodwin et al., 2018).

2 $$ {\dot{E}_{{\rm{ch}}}} = \dot{F}\ \ {e_{{\rm{ch}}}} = \dot{F}\ \left[ {\mathop \sum \limits_i^N {x_i}e_{{\rm{ch}},i}^0 + \ R{T_0}\mathop \sum \limits_i^N {x_i}\ ln{x_i}} \right]. $$

Where ${\dot{E}_{{\rm{ch}}}}$ is the chemical exergy flow, ${e_{{\rm{ch}}}}$the specific molar chemical exergy, ${x_i}$ the molar fraction of substance *i*, and $e_{{\rm{ch}},i}^0$ is the standard molar chemical exergy of substance *i*. Values for $e_{{\rm{ch}},i}^0$ are tabulated in several studies and in this work they are taken from Szargut’s original table of specific chemical exergies (Szargut et al., 1988). The full table of values, with other important parameters can be found in the Supporting Information.

Equations (3) and (4) show the electricity and heat exergy calculations. Electricity is pure work and thus equal to exergy, while heat is scaled by the Carnot factor.

3 $$ {\dot{E}_{{\rm{el}}}} = {\dot{E}_{{\rm{comp}}}} $$

4 $$ {\dot{E}_Q} = \left( {1 - \frac{{{T_0}}}{{{T_{\rm{h}}}}}} \right)\ {\dot{Q}_{{\rm{loss}}}} $$

Where ${\dot{E}_{{\rm{el}}}}$ is electrical exergy flow, the compressor electricity flow, ${\dot{E}_Q}$ the heat loss exergy flow, ${\dot{Q}_{{\rm{loss}}}}$ the heat loss energy flow, and ${T_{\rm{h}}}$ the temperature of the heat loss.

### System determination

The exergy equations outlined in Equations (1)–(4) inform the data requirements: flowrate, temperature, pressure, and composition of input and output flows as well as electricity and heat inputs and outputs over time. The availability of this data will affect the detail and resolution of the system boundary chosen for the analysis. Ideally, the resolution would be high enough to attribute exergy losses and destruction to every equipment in the plant. However, unlike modeled data typically used in exergy studies, cost implications mean that real industrial flows often lack metering of one or more of these variables for one or more process streams. Therefore, the analysis is initially done at the SMR plant scale.

**FIGURE 1:**
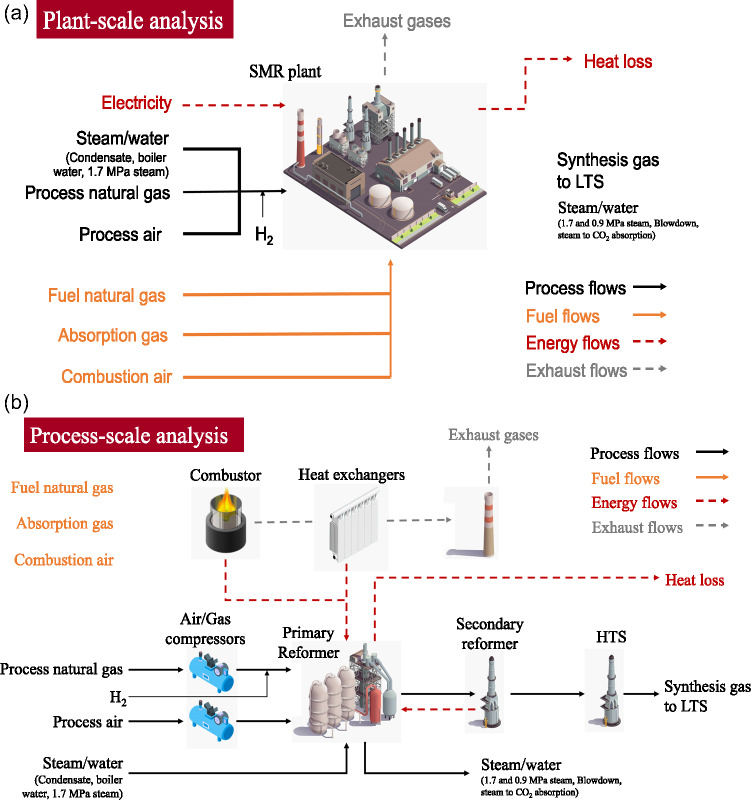
System boundary and key input and output flows for the (a) steam methane reforming (SMR) – high-temperature shift (HTS) plant-scale analysis and (b) the process-scale analysis. In (b), SMR consists of the majority of the entire SMR plant, including the primary reformer, feed heat exchangers, and steam-raising vessels but excludes the secondary reformer and the combustor. Images from Macrovector (n.d.)

Metering devices in the SMR plant and high-temperature shift (HTS) reactor are ubiquitous, so the system boundary is drawn around those processes. As shown in Figure 1a, the plant converts process natural gas, steam, and air into synthesis gas with auxiliary steam and water production, while the combustion of fuel waste and natural gas drives the process. Electricity powers the process gas and air compressors with some heat losses arising from the main reactor vessel as well.

The analysis is also performed at the process scale. The combined SMR-HTS plant is split into its constituent processes in as much detail as the data sensors will allow, as shown in Figure 1b. The fuel streams flow into the combustor with the exhaust gases passing through a heat exchange section before being released to the atmosphere. The process flows are compressed before entering the first section, which is named SMR as it includes the majority of the SMR equipment, notably the primary reformer and the feed heat exchangers. Then the synthesis gas flows through the secondary reformer and the HTS reactor before proceeding to the low-temperature shift (LTS) reactor.

### Raw data filtering and cleaning

The data provided included measurements of 311 sensors over 2 years, totaling 327 million data points so, given the resolution of the analysis, not all sensor data are required. To filter the data, the required material flows are traced in hybrid process flow and piping and instrumentation diagrams (PFDs and P&IDs). The relevant sensor tags are identified and used to filter the original datasets while metadata denoting the stream the sensor is measuring is also attached. This process amalgamates the values measured for every sensor with the stream the measurements belong to and allows for a data structure akin to that used by process simulators when presenting stream data. We employed standard statistical techniques to check for zero and low-variance variables, non-numerical entries, multi-format timestamps, and other common issues with real industrial data. The minute-level data has been aggregated to daily level to reduce computational costs and the time taken to perform the analysis.

**FIGURE 2:**
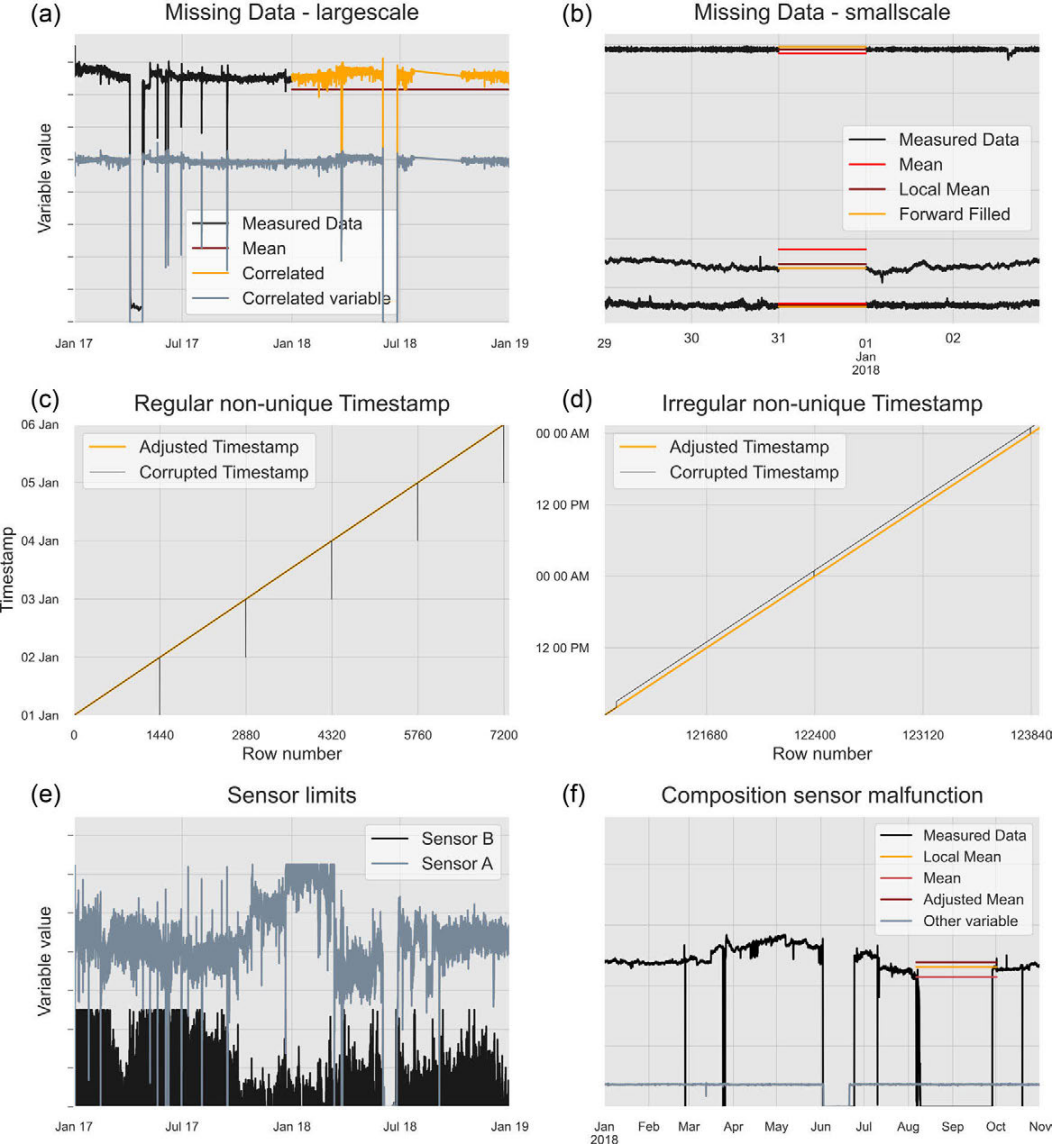
Data cleaning issue types. (a) An example of a large subset of a sensor’s values missing and two imputation strategies based on a mean and adjusted mean approach. (b) Example of a small subset of a sensor’s values missing and an imputation strategy based on forward filling. (c) Regular examples of duplicated timestamps, 1 min every day. (d) Irregular examples of duplicated timestamps, an entire hour missing. (e) An example of two sensors with upper limit in the measuring range. (f) Composition analysers malfunctioning and imputation strategies based on different mean values. Data underlying this figure are available in Supporting Information S2

Having filtered the data, it is necessary to clean it. Figure 2 presents a series of different data issues faced with this dataset. A decision tree in the Supporting Information details the decision of how to correct each type of these issues. Figure 2a,b illustrates two cases where data is missing from a particular sensor, that is, there is no value, and imputation techniques are required. In the first case, one variable, an output steam temperature, is missing 2018 data. To preserve the dynamic nature of the data, we estimate the 2018 values using a linear regression based on the best correlated variable with this one, the stream’s pressure (see Supporting Information for details and comparison with other imputation methods). In the second case, shorter timeframes of data are missing, such as the illustrated missing 31st of December. As shown by the red line, mean-based imputation would once again yield locally unrepresentative values, noticeably different to the days surrounding them. A “local” mean (one day before and after) of the data in the surrounding days does not guarantee a representative value either so a forward fill approach is chosen, which fills based on the last measured value.

Figure 2c,d shows the effect of duplicated timestamps in the measurements. The timestamp is plotted against the row number and for a clean, continuously increasing timestamp we expect a straight line with no sudden increases or decreases. Duplicate timestamps can impact various operations such as data aggregation and trend calculations while also leading to gaps in the data. Regular timestamp duplications, such as every day 11:54 p.m. would be replaced with 12 a.m. (shown as the black dips in Figure 2c), are easy to fix. However, Figure 2d shows irregular changes such as a jump from 2 a.m. to 3 a.m. or 1 a.m. being replaced by 12 a.m. as shown in black. This combination of regular and irregular duplication requires a complete re-indexing of the data with unique timestamps covering 2017 and 2018 with a frequency of 1 min. This adjustment is shown in both figures with the orange line.

Figure 2e illustrates an issue present in a few of the measured variables, predominantly flowrates: values have an upper ceiling. It is impossible to tell if these variables are limited by the control system or the sensor cannot measure higher limits. Thus, there is no safe way of imputing actual values and no adjustment is made to the data. Finally, Figure 2f shows an example of corrupt data. Between August and September, the composition measurements for CO_2_, CO, and CH_4_ after two reformers were zero. No variables were correlated enough with the compositions to follow such an imputation strategy. Imputing with the mean or an adjusted mean leads to unrepresentative values for that time period. Instead, we calculate a local mean using data around the corrupt period (see Supporting Information for details).

Composition measurements are also zero in most of June. Other variables, one of which is plotted, exhibit a concurrent similar drop, also evident in Figure 2e, indicating a plant shutdown; so there is no need for correction. This exemplifies the need for context when filling missing or zero values in industrial measurements as not all of these should be rectified, especially when they represent reality.

### Data reconciliation

Following data cleaning, it is necessary to estimate any unmetered variables for both material and energy flows.

#### Material flows

The guiding principle for this data reconciliation procedure is that the only extensive variable, flowrate, should be estimated in relation to other metered flowrates as, when the site’s production levels fluctuate, all flowrates are likely to fluctuate in tandem. For intensive variables like pressure and temperature, the estimation should ideally also be based on metered data; for example, T and P of input combustion air, which is unmetered, is set equal to the metered T and P of the process air. When this is not possible (especially with composition data), values are used from a static model representing normal operation provided by the plant operators. A summary of the estimated values is shown in Table Table 1 while more detailed is provided in the Supporting Information.

**TABLE 1:** Summary of estimated variable values that are not metered. Empty cells indicate that metered data is available. Streams shown in Figure 1 but not in this table are fully metered. Composition column shows main substances. Gas analysis was provided by the plant operators using gas chromatography

Stream	Temperature	Pressure	Flow	Composition	Source
Boiler water input		44 bar		Pure H_2_O	Model
Condensate input		40 bar		99.9% H_2_O	Model
Blowdown	245^o^C	36.7 bar	Boiler tank input – Boiler tank output	Pure H_2_O	Balancing - Model
Hydrogen input	20^o^C			18% H_2_, 78% CO_2_	Assumption - Model
Absorption gas (AG) input	30^o^C			66% N_2_, 15% CH_4_, 13% H_2_	Model
Combustion air	Atmospheric temperature	1.01325 bar	13.44*(Fuel gas + AG)	75% N_2_, 23% O_2_	Balancing – Model
Exhaust gases			Fuel gas + AG + Combustion air	71% N_2_, 13% CO_2_, 13% H_2_O	Balancing
All steam inputs/outputs				Pure H_2_O	Model
Process natural gas				91% CH_4_, 6% N_2_	Gas analysis
Fuel natural gas				86% CH_4_, 10% N_2_	Gas analysis
Syngas to LTS		Pressure drop	Sum of process inputs	CH_4_, CO_2_, CO metered. Rest are estimating from reaction modeling	Reaction modeling

#### Energy flows

The site has two main electricity inputs for the process air and process natural gas compressors. The electricity use of those compressors is estimated as their rated power scaled by the ratio of the actual fluid flow rate to the rated flow rate, as shown in Equation (5). There is also heat lost from the primary reformer process, which is estimated from baseline heat losses in the model and scaled as a ratio of exhaust gas flow to “rated” exhaust gas flow value from the model, as shown in Equation (6):

5 $$ \dot{E}{l_{{\rm{comp}}}} = \dot{E}{l_{{\rm{comp}},{\rm{rated}}}}\ \frac{{\dot{F}}}{{{{\dot{F}}_{{\rm{rated}}}}}}, $$

6 $$ {\dot{Q}_{{\rm{loss}}}} = {\dot{Q}_{{\rm{loss}},{\rm{model}}}}\ \frac{{{{\dot{F}}_{{\rm{exhaust\ gas}}}}}}{{{{\dot{F}}_{{\rm{exhaust\ gas}},{\rm{rated}}}}}}. $$

### Efficiency definitions

The exergetic performance of an industrial system at any scale can be assessed by the evaluation of its exergy (resource) efficiency:

7 $$ \eta \ = \frac{{\mathop \sum \nolimits_i {{\dot{E}}_{{\rm{out}},i}}}}{{\mathop \sum \nolimits_i {{\dot{E}}_{{\rm{in}},i}}}}\ = \ 1 - \frac{{{{\dot{E}}_{\rm{d}}} + \mathop \sum \nolimits_i {{\dot{E}}_{{\rm{l}},i}}}}{{\mathop \sum \nolimits_i {{\dot{E}}_{{\rm{in}},i}}}}. $$

Where $\eta$ is the conventional exergy efficiency, ${\dot{E}_{{\rm{out}},i}}$ and ${\dot{E}_{{\rm{in}},i}}$ are the useful output and input streams, respectively, ${\dot{E}_{\rm{d}}}$ is the exergy destroyed, and ${\dot{E}_{{\rm{l}},i}}$ is the exergy lost in the form of waste material or heat. The exergy loss term denotes exergy associated with waste heat or materials whereas the exergy destruction term arises from irreversibilities within the process that are not associated with a flow of matter. This conventional efficiency definition can lead to high efficiency values as it does not take into account the fact that, often, large amounts of exergy inputs are not part of any transformation in the system but rather flow through unaltered (Kirova-Yordanova, 2004). The larger the proportion of input exergy that just flows through the system untransformed (e.g., exergy associated with inert gases such as nitrogen or chemical exergy that does not participate in heat exchange processes), the smaller the ratio of exergy destruction to exergy input, leading to higher efficiencies. See the Supporting Information for a more detailed and visual explanation. A reformulated efficiency definition that compares the net product of a system to the net fuel consumed while also taking into account the purpose of the system addresses this issue. This definition is known as the fuel-product efficiency (Bejan et al., 1996; Tsatsaronis, 1993). In this work, due to the resolution of the analysis and the way equipment is grouped together into processes, determining the purpose of the individual systems is not straightforward. Instead, we use a parallel approach where the untransformed exergy known as “transiting exergy” is taken into account by the efficiency definition (Flórez-Orrego & de Oliveira Junior, 2016):

8 $$ {\eta _{{\rm{tr}}}} = \frac{{\mathop \sum \nolimits_i {{\dot{E}}_{{\rm{out}},i}} - {{\dot{E}}^{{\rm{tr}}}}}}{{\mathop \sum \nolimits_i {{\dot{E}}_{{\rm{in}},i}} - {{\dot{E}}^{{\rm{tr}}}}}}\ = \frac{{\mathop \sum \nolimits_i {{\dot{E}}_{{\rm{out}},i}} - \left( {\dot{E}_{{\rm{ph}}}^{{\rm{tr}}} + \dot{E}_{{\rm{ch}}}^{{\rm{tr}}}} \right)}}{{\mathop \sum \nolimits_i {{\dot{E}}_{{\rm{in}},i}} - \left( {\dot{E}_{{\rm{ph}}}^{{\rm{tr}}} + \dot{E}_{{\rm{ch}}}^{{\rm{tr}}}} \right)}},{\rm{\ }} $$

9 $$ \dot{E}_{{\rm{ph}},{\rm{ch}}}^{{\rm{tr}}} = \ min\left[ {\mathop \sum \limits_{{\rm{in}}} \dot{E}{\,_{{\rm{ph}},{\rm{ch}}}}_{{\rm{in}}},\mathop \sum \limits_{{\rm{out}}} \dot{E}{\,_{{\rm{ph}},{\rm{ch}}}}_{{\rm{out}}}} \right]. $$

Where ${\dot{E}^{{\rm{tr}}}}$ is the transit exergy and $\dot{E}_{{\rm{ph}},{\rm{ch}}}^{{\rm{tr}}}$ denotes its physical and chemical components. They are estimated as the minimum of the input and output chemical and physical exergy flows. While the fuel-product definition is more widely used, in a recent study on an SMR plant we showed that in practice, the transit and fuel-product efficiency definitions do not yield significantly different results (Michalakakis et al., 2019). More advanced exergetic methods develop algorithms to split exergy destruction and loss into avoidable and unavoidable components, thus providing even more detail into the potential resource efficiency opportunities (Kelly et al., 2009). These methods rely on calculating ideal efficiencies for different systems to calculate the unavoidable inefficiency but are more developed for thermal systems such as power plants, and are therefore not employed in this study.

### Resource visualization

Sankey diagrams are commonly used for visualizing resource flows in complex systems (Schmidt, 2008). They are chosen over Grassmann diagrams, traditionally used in exergy analysis, as assigning a virtual “flow” to exergy destruction is more visually impactful than a decrease in thickness and the audience can more easily identify which areas of the plant contribute most to exergy destruction and loss. The Sankey diagrams are drawn with the open-source Python-based floWeaver (Lupton & Allwood, 2017).

## RESULTS

This section presents the results of the exergy analysis: resource flow visualization in Sankey diagrams and dynamic results for key exergy indicators at the plant and process scale.

### Flow visualization

**FIGURE 3:**
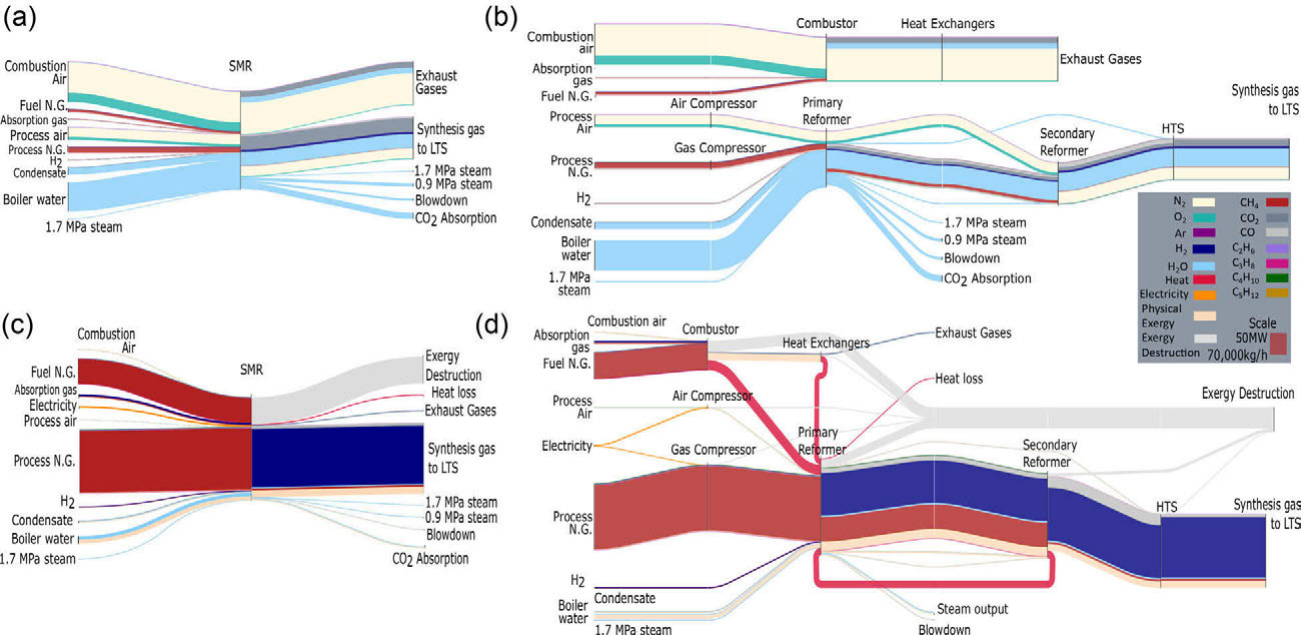
Sankey diagrams visualizing flows in the system boundary analyzed. (a) Mass diagram of plant-scale analysis. (b) Mass diagram of the process-scale analysis. (c) Exergy diagram of the plant-scale analysis. (d) Exergy diagram of the process-scale analysis. Data underlying this figure are available in Supporting Information S2

Figure 3 pictures indicate mass and exergy Sankey diagrams for both the plant-scale and the process-scale analyses. These diagrams depict the flows at the first day of operation and serve as indicative visualizations to highlight the highest exergy value flows through the plant and indicate the location of the highest exergy losses.

Figure 3a,b visualizes the material flows through the plant in mass units: for the plant and process-scale system boundaries, respectively. In the top section of the Sankey diagrams, the fuel natural gas is combusted with some additional post-absorption fuel gas, to provide heat to the SMR reaction. The exhaust gases provide heat to process streams such as natural gas, air, and steam through heat exchangers. In the bottom, process air and natural gas are compressed by electricity-driven compressors, while steam and water are fed to the process. Methane partially converts to hydrogen in the primary reformer with side products of carbon monoxide and carbon dioxide. In the secondary reformer and the HTS reactor, the methane and carbon monoxide are gradually converted to carbon dioxide.

Figure 3c,d illustrates the exergy flows for the plant and process-scale analyses, respectively. While flows such as air and steam dominate the mass diagram, these are much less prominent in the exergy diagram where methane and hydrogen flows dominate. These are not only the most energetically valuable flows but also, given that natural gas is the main operating cost of SMR processes (Zhang et al., 2020) and hydrogen the main product, the most financially valuable as well. This correlation supports the argument that exergy better reflects a resource’s value. The heat provided by the fuel burning in the combustors is shown in pink. The exhaust gases’ exergy is mainly made up of physical exergy due to their high temperature, which is also used to provide heat to the main process route. The secondary reformer provides some heat to the primary reformer. The light gray flows represent the exergy destruction occurring in each of the processes. The combustor is responsible for the highest rate of exergy destruction, followed by the primary reformer while the heat exchangers, the secondary reformer, and the HTS reactor contribute smaller amounts. Finally, exergy lost via heat losses and the exhaust gases is negligible compared to the much higher exergy destruction. This observation supports the claim that exergy analysis can identify more accurately the true location and magnitude of resource loss in industry.

### Plant-scale analysis

**FIGURE 4:**
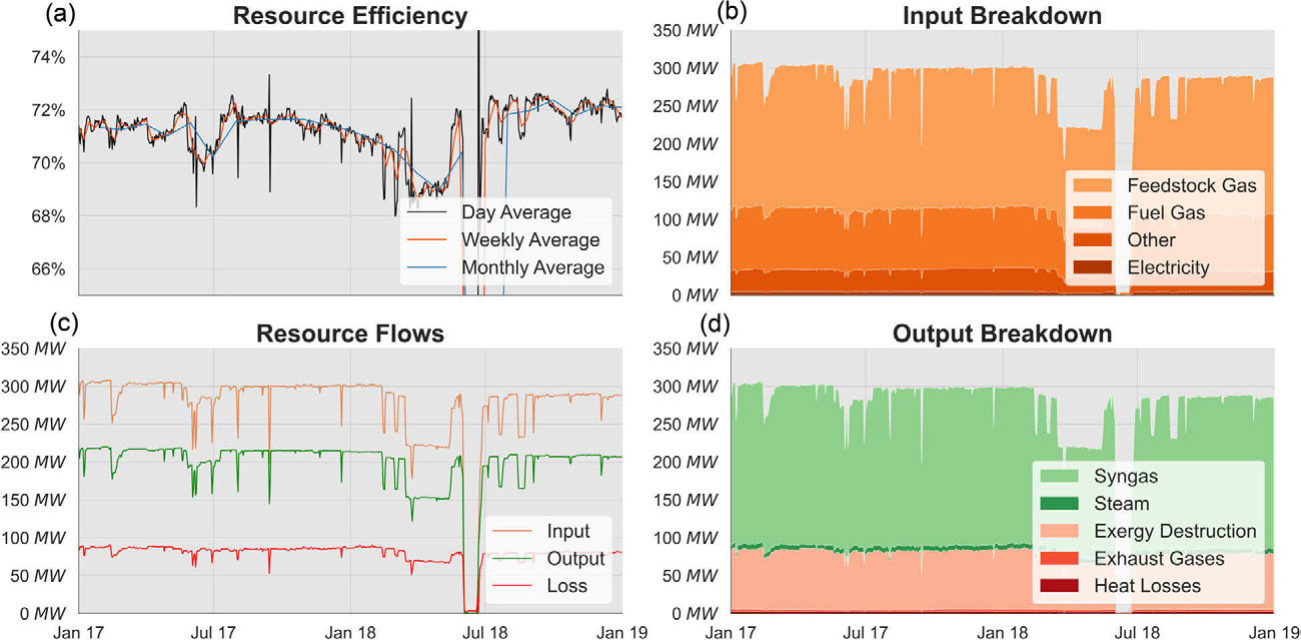
Plant-scale exergy analysis results. (a) Resource efficiency averaged daily, weekly, and monthly. (b) Input flow breakdown. (c) Exergy input, output, and loss. (d) Output flow breakdown. Where not stated, data is averaged daily. Data underlying this figure are available in Supporting Information S2

Figure 4 illustrates the results of the plant-scale analysis. Figure 4a,c depicts the resource efficiency and the exergy resource flows (input, output, and loss) varying over the 2 years of operation measured in the data collected. Figure 4b,d breaks down the total input, useful output, and loss to the constituent flows in shades orange, green, and red, respectively. The exergetic performance of the plant and the exergy flows are relatively stable: efficiency fluctuates in the range of 68–73% while the inputs, useful outputs, and loss vary around 300, 220, and 80 MW, respectively. Production ramp-downs occur in April and May 2018 while a prolonged shutdown in June 2018 leads to extreme results in the exergy efficiency.

Feedstock gas makes up the majority of the exergy input with approximately 170 MW, followed by the fuel gas at 75 MW. Steam and air inputs make up most of the remainder with electricity also providing around 4 MW. Syngas at around 190 MW accounts for the overwhelming majority of the useful output exergy with auxiliary steam making up the rest. Exergy destruction at 72 MW is the major cause of loss with external losses such as exhaust gases and heat losses accounting for another 5 MW of loss.

### Process-scale analysis

**FIGURE 5:**
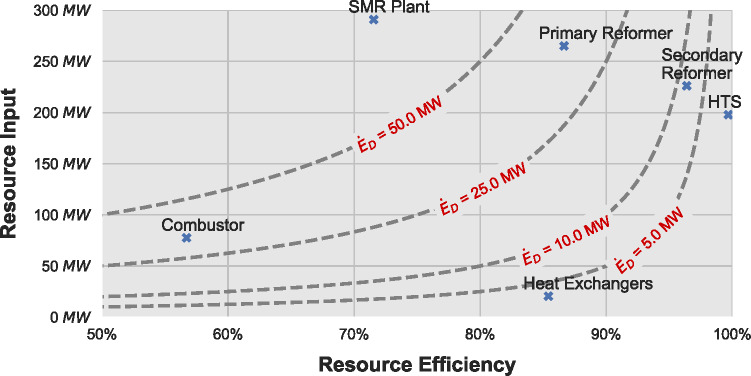
Resource input versus resource efficiency for the SMR plant and its constituent processes. The dotted lines represent equal exergy destruction. Data underlying this figure are available in Supporting Information S2

A summary plot of the process and plant average exergy input, efficiency, and loss is shown in Figure 5. The dotted lines represent equal amounts of exergy destruction, which increases toward the top left of the figure. The overall SMR plant is also included for comparison purposes. The HTS reactor and the secondary reformer have the highest efficiency with 99% and 96%, respectively, indicating that the singular purpose of these processes and the low extent of reaction compared to the overall amount of substance entering them leads to low irreversibilities and exergy destruction. The heat exchangers have an efficiency of 85% with a low resource input of 20 MW, which means they present with a small amount of exergy destruction averaging 3 MW. Finally, the primary reformer and the combustor are responsible for the highest amount of exergy destruction with an average of 35 and 33 MW, respectively. This is attributed to the primary reformer’s high exergy input at 265 MW and the combustor’s low efficiency at 57%. The multiple heat exchange, mixing, and reaction processes taking place in the primary reformer and the notoriously inefficient combustion reaction can explain this high exergy destruction.

**FIGURE 6:**
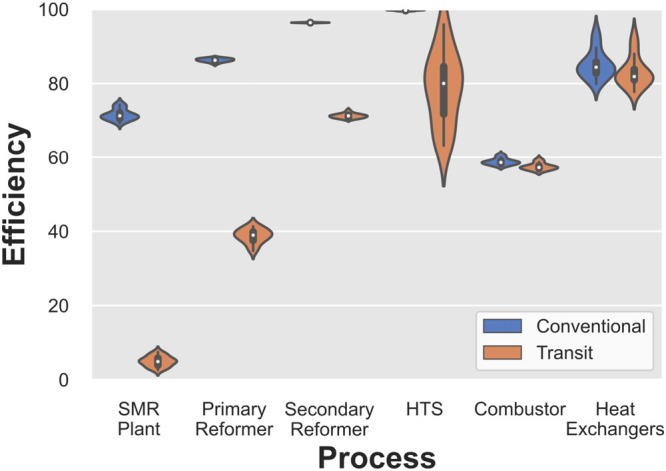
Violin plot of conventional versus transit efficiency definitions for the SMR plant and its constituent processes. Data underlying this figure are available in Supporting Information S2

Figure 6 compares conventional exergy efficiency to transit efficiency, which accounts for untransformed exergy flows that skew conventional efficiency values higher. The violin plot shows the distribution of efficiency values and compares how the two values vary for every process. Transit efficiency is lower than the conventional efficiency for all processes and the plant, indicating that the reformulated efficiency definition’s suitability is more representative of the system’s real performance. The discrepancy between the two efficiencies is less pronounced when the transit exergy flowing through the system is low, which is the case in the combustor and heat exchanger where the majority of the input exergy takes part in some transformation. Conversely, in the reactor vessels where the extents of reaction are low compared to the overall amount of substances entering the vessels, the discrepancy between the two efficiencies is higher. Counter-intuitively, this is also the case with the SMR plant as a whole, where the transit efficiency is approximately 5% as opposed to a conventional efficiency of 83%.

Second, transit efficiencies are more widely distributed than the conventional efficiencies. This suggests that a seemingly stable performance according to the conventional efficiency metric is actually more variable and real performance is more sensitive to operating conditions and changes to the plant.

**FIGURE 7:**
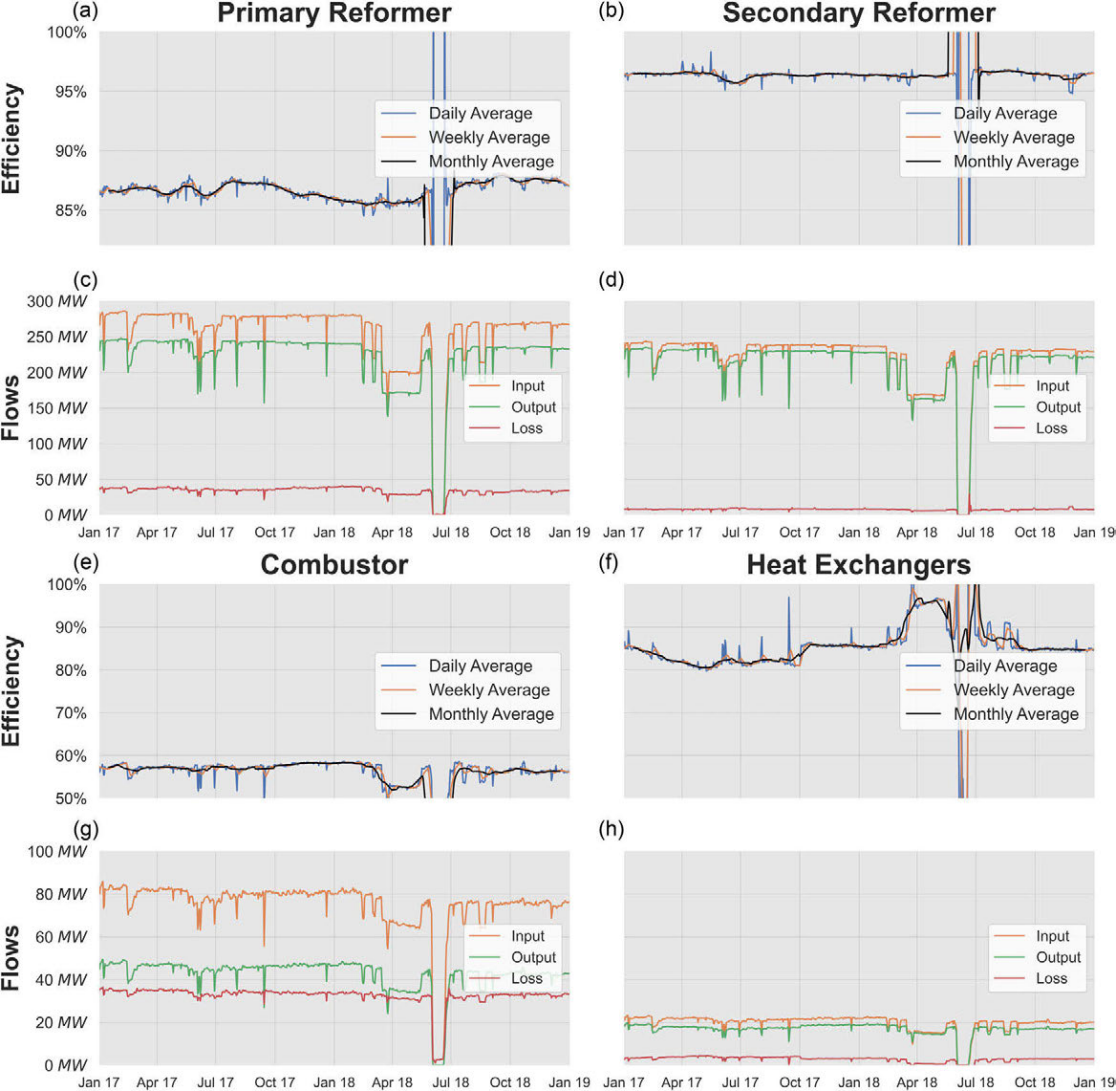
Process-scale exergy analysis results. (a,b,e,f) Resource efficiency averaged daily, weekly, and monthly for the primary reformer, secondary reformer, combustor, and heat exchangers. (c,d,g,h) Exergy input, output, and loss. Where not stated, data is averaged daily. Data underlying this figure are available in Supporting Information S2

Finally, Figure 7, visualizes the exergy indicators for the processes over time. The HTS reactor is not included in Figure 7 due to its low exergy loss, as can be seen in Figures 3c and 5. As with the plant-scale analysis, sporadic production ramp-downs can be observed with the period from March until May 2018 being the most prominent. The complete shutdown in June 2018 is also visible leading to nonsensical resource efficiency and flow results; however, that only affects a small portion of the results so can be ignored.

Most process efficiencies are stable, staying within 5% of their average. The heat exchangers are the exception with a wider range of about 10% around their mean, mainly due to an increase in efficiency during a production ramp-down. However, this relationship between production rate and efficiency is usually the inverse: when production rate decreases, efficiency decreases as well. This is observed during June 2017 where efficiency decreased in the combustor, primary, and secondary reformers, while the March–May 2018 ramp-down saw an even larger efficiency drop in the combustor. While the exergy input and output decrease during these ramp-downs, exergy loss does not decrease at a 1:1 ratio with them, indicating that it is inelastic and therefore, leading to higher production rates driving higher production efficiency.

## DISCUSSION

Efficiency improvement approaches can generally involve retrofitting plants with improvement projects, “finetuning” performance by optimizing process parameters and recovering by-products and waste to be used in other industries (Leigh & Li, 2015). Exergy analysis can help identify opportunities for all three of these avenues, by quantifying the wasted resources and identifying inefficient hotspots. In the case of ammonia production, however, industrial decision-makers only have two “levers” to control when improving performance: retrofit improvements and “finetuning” performance. This analysis indicates where in the SMR plant the highest inefficiencies and exergy losses are: the primary reformer’s and the combustor’s high exergy losses point to resource saving opportunities. The secondary reformer and the heat exchangers present smaller losses. While the combustor has the lowest conventional efficiency, it is the primary reformer process that presents the lowest transit efficiency indicating that when transit exergy is taken into account it is not as efficient. This reflects the fact that a number of processes are included in the primary reformer system boundary in this analysis due to lack of data that, if resolved, could lead to even more granular analysis.

This dynamic analysis can aid plant decision-makers by setting a performance benchmark over time for the processes. The SMR plant achieved efficiency of 73% several times in the analysis period but averages around 71%; the implication being that around 6 MW of resources could be saved just by reaching already achieved performance. In a real-time implementation of this exergy performance metric, plant operators can be aware of what decisions and parameters such as feedstock or output levels lead to improved performance and adjust the plant accordingly.

Not all losses can be recovered and advanced exergy analyses methods that identify the unavoidable component of exergy destruction can provide a more accurate estimation of the potential savings; however, they rely on estimates of thermodynamic limits for processes that are much better developed for power plants so these methods have yet to be applied to chemical plants (Kelly et al., 2009).

Whilst no other study has performed dynamic exergy analysis on an SMR plant, we can compare our results against studies on models. The most comprehensive recent study found conventional (and transit) efficiencies: 75% (74%), 97% (76%), and 96% (53%) for the combustors, primary, and secondary reformer, respectively (Flórez-Orrego & de Oliveira Junior, 2016). Our results are consistently below the literature values, as expected for a real plant versus a theoretical one; this shows that real data provide a less optimistic view of industrial performance than model data typically used. We also find similar discrepancies between the conventional and transit efficiencies for the reformers and a lack of significant difference in those values for the combustor.

### Data requirements

The ubiquity of meters and sensors around the site heavily influences the exergy analysis: first, the boundary of the analysis is ideally drawn around the best-metered sections of the site and secondly, the scale of the analysis needs to be high enough so that all relevant variables are metered. This minimizes the need to estimate or model variables, reducing uncertainty and potential errors in the analysis. However, restricting the boundary and performing the analysis at a larger scale leads to resolution loss and exclusion of parts of the site, reducing potential insights. In particular, higher resolutions can attribute losses to specific equipment guiding improvement projects and enable the evaluation of fuel-product efficiency.

Real data collected from sensors may often present with outlier values, due to sensor malfunction, failure, or other events (Gaddam et al., 2020). In this analysis, averaging minute data to daily largely deals with these outliers. Uncertainty also comes from assumptions about unmetered data. In many cases, we used mass balancing and flow ratios (e.g., fuel-to-air ratio for the combustor) to estimate dynamic flows. However, this assumes no build-up in vessels and steady flow ratios: this might not always be true. For instance, while a change in air flow would barely affect the exergy input due to the low exergy of air, it would affect the combustion reaction and thus the heat production and exhaust gas composition. Comparing Table Table 1 with the exergy flows visualized in Figure 3, assumptions and model data were used for variables and streams that have low exergy associated with them, steam/water flows, exhaust gases, and air supply, for example. The impact of the assumptions on the overall results is therefore likely to be low. Variables for the high-exergy flows such as the natural gas feed and fuel and the syngas product are mostly estimated using flow balancing and real data that are more realistic than constant values. The assumption that the composition of feed and fuel natural gas remains constant introduces some error, however, but can be rectified by taking more frequent analysis samples.

While only one variable was missing half the data that was filled with linear regression, real data might have randomly distributed missing data for several variables. In that case, more sophisticated machine-learning approaches are excellent for imputing missing data (Nagashima & Kato, 2020; Gruenwald et al., 2007).

Continuous process typically monitor data at the same frequency but batch processes may have material flows metered per batch and daily energy flows. This requires further data reconciliation and resampling. Finally, the thermodynamic package Cantera provides an excellent implementation of thermodynamics models in Python but has not yet implemented the Peng–Robinson equation of state that is often used for its balance between simplicity and data accuracy (Peng & Robinson, 1976).

### Potential of dynamic industrial exergy analysis

An analysis comparing different parts of the site will yield the best results when having a high resolution that will allow for loss attribution to as many equipment pieces and loss mechanisms as possible. This is not always feasible with real data and there is a trade-off between higher resolution and higher fidelity to measured data. Industry 4.0 and cheaper, wireless sensors for industrial purposes provide two potential opportunities. Firstly, low-cost wireless modern sensors can fill missing data to create higher-resolution exergy pictures with broader scope and increased fidelity to real conditions. Second, real-time monitoring and the coupling of exergy with sensors can inform operator control decisions through real-time exergy analysis.

## CONCLUSIONS

We applied exergy analysis on real data collected from an SMR plant in an ammonia site. Exergy analyses in the literature are typically performed at system level or on simulations of industrial sites. These studies inform policymakers and process designers, providing insight and informing process design decisions. However, industrial decision-makers at the site level also have influence over industrial performance, through identifying improvement opportunities and operations finetuning.

The number of sensors in a site can affect the boundary and resolution of the exergy analysis, in turn affecting the insight that can be obtained. Industrial sensor data present unique data cleaning challenges and require a degree of engineering intuition to perform tasks such as data imputation. Modeling techniques including material balances and reaction modeling are also required to estimate variables needed for the analysis.

This analysis was applied to an ammonia site, one of the largest chemicals by volume, energy consumption, and GHG emissions (FAO, 2020). However, it is applicable in any industrial setting, particularly where both material and energy resources are used, for example, steel. Increasing numbers of cheaper wireless sensors can improve the resolution, scope, and accuracy of industrial exergy analysis, while modern software can enable real-time performance monitoring. Future work is planned to rigorously correlate specific operating parameters and variables to the exergy, cost, and environmental performance of the plant that can inform plant operators about specific levers they can “pull on” to finetune performance. This will also investigate whether improved resource efficiency leads to reduced emissions.

## Supplementary Information

**Supporting Information S1**: This supporting information S1 provides information such as equations, data and constants used in the methodology. This includes the methodology to estimate variables that weren.t metered and the exergy methodology. (DOCX 753 KB)

**Supporting Information S2**: This supporting information S2 provides the underlying data plotted in Figures 2-7 in the manuscript. Figures 2,4-7 can be visualised with any software. Figure 3 is the Sankey diagram data which can be visualised with the open-source Python library floWeaver or with eSankey! which is a more intuitive software for the beginner. (XLSX 91.3 MB)

## Data Availability

Research data are not shared.
