# Supplementary material for: Dynamic exergy analysis: From industrial data to exergy flows
Source: J Ind Ecol. 2021 Jul 19;26(1):12–26. doi: 10.1111/jiec.13168 (PMC13090283; doi:10.1111/jiec.13168)
Supplement: Supplementary file 1 — Supporting Information S1: This supporting information S1 provides information such as equations, data and constants used in the methodology. This includes the methodology to estimate variables that weren.t metered and the exergy methodology. (DOCX 753 KB) [file 44498_2022_2601002_MOESM1_ESM.doc]

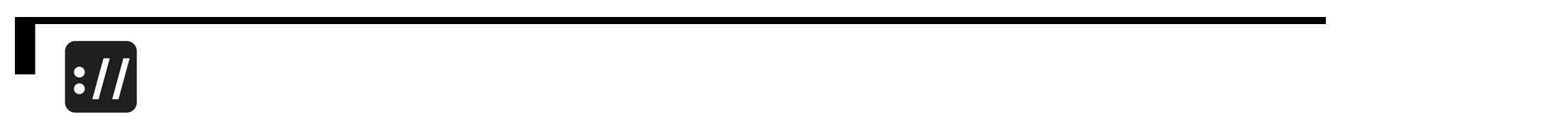


SUPPORTING INFORMATION FOR:

Michalakakis, C., Cullen, J.M. (2021.). Dynamic Exergy Analysis: From Industrial Data to Exergy Flows. *Journal of Industrial Ecology.*

**
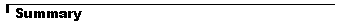
**

This supporting information S1 provides information such as equations, data and constants used in the methodology. This includes the methodology to estimate variables that weren’t metered and the exergy methodology. Included in the submission but separate from this SI document is a .xls file with the underlying data for all figures in the manuscript.


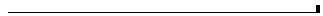


## Sensor overview

This section provides an overview of the number and type of sensors from which data was collected and used in the analysis. Gas analysis is performed with gas chromatography to find composition of CO, CO_2_, CH_4_ after key equipment (primary/secondary reformer, HTS, LTS etc.) as well as trace amounts of H_2_, N_2_ and Ar at the recycle flow. Temperature is always measured in ^o^C and is placed throughout the plant while pressure is measured in bar, kPa and MPa and mainly measures pressure with some instances of pressure drop. Flow is measured in kg/h, m^3^/h and Nm^3^/h and the sensors are placed at key locations.

Table 1: Sensor types and numbers from which data was collected

|  | | **Total sensors** | **Sensors used at plant-scale** | **Sensors used at process-scale** |
| --- | --- | --- | --- | --- |
| **Temperature** | | 122 | 10 | 15 |
| **Flow** | Mass | 27 | 7 | 9 |
|  | Volume | 32 | 6 | 7 |
| **Pressure** | | 74 | 9 | 12 |
| **Gas analysis** | | 21 | 3 | 9 |
| **Other (levels, flow ratios, fan speeds etc.)** | | 35 | - | - |

## Data Processing

This section outlines procedures for data processing (cleaning, exergy calculations etc.).

### Unit Alignment

Pressures are measured with different units so they are all aligned to bar from MPa and Pa.

Flows are typically measured in kg/h and Nm^3^/h. One condensate flow is measured in m^3^/h. The volumetric flow rates are converted to mass flow rates according to the following equations:

| 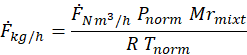 |
| --- |
| 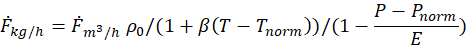 |


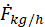
 is the mass flow rate in kg/h and
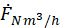
 and
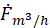
 are the volumetric flow rates is Nm^3^/h and m^3^/h respectively.
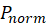
 and
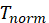
 are the normal pressure and temperature taken as 1.01325 kPa and 0^o^C respectively.
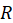
 is the universal constant whose value is set at 8.3144598 kPa m^3^/(K kmol).
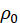
 is the density of water at 1.01325 kPa and 0^o^C, 999.8 kg/m^3^.
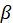
 is the volumetric thermal expansion coefficient of water whose value 0.00068 K^-1^ is taken at 90 ^o^C, the midpoint between
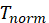
 and the typical temperature of the condensate, around 180 ^o^C (Yaws, 2012).
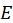
 is the bulk modulus of water taken at 90 ^o^C, which is 2.15x10^9^ Pa.
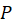
 and T are the pressure and temperature of the condensate. Temperature is measured by a temperature sensor and pressure is taken as 40 bar based on the static data.
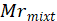
 is the molar weight of the mixture, calculated as a weighted average of the molar weights of the individual substances in the stream.

### Data Cleaning

The strategies followed to reconcile missing or un-metered data is summarised below:


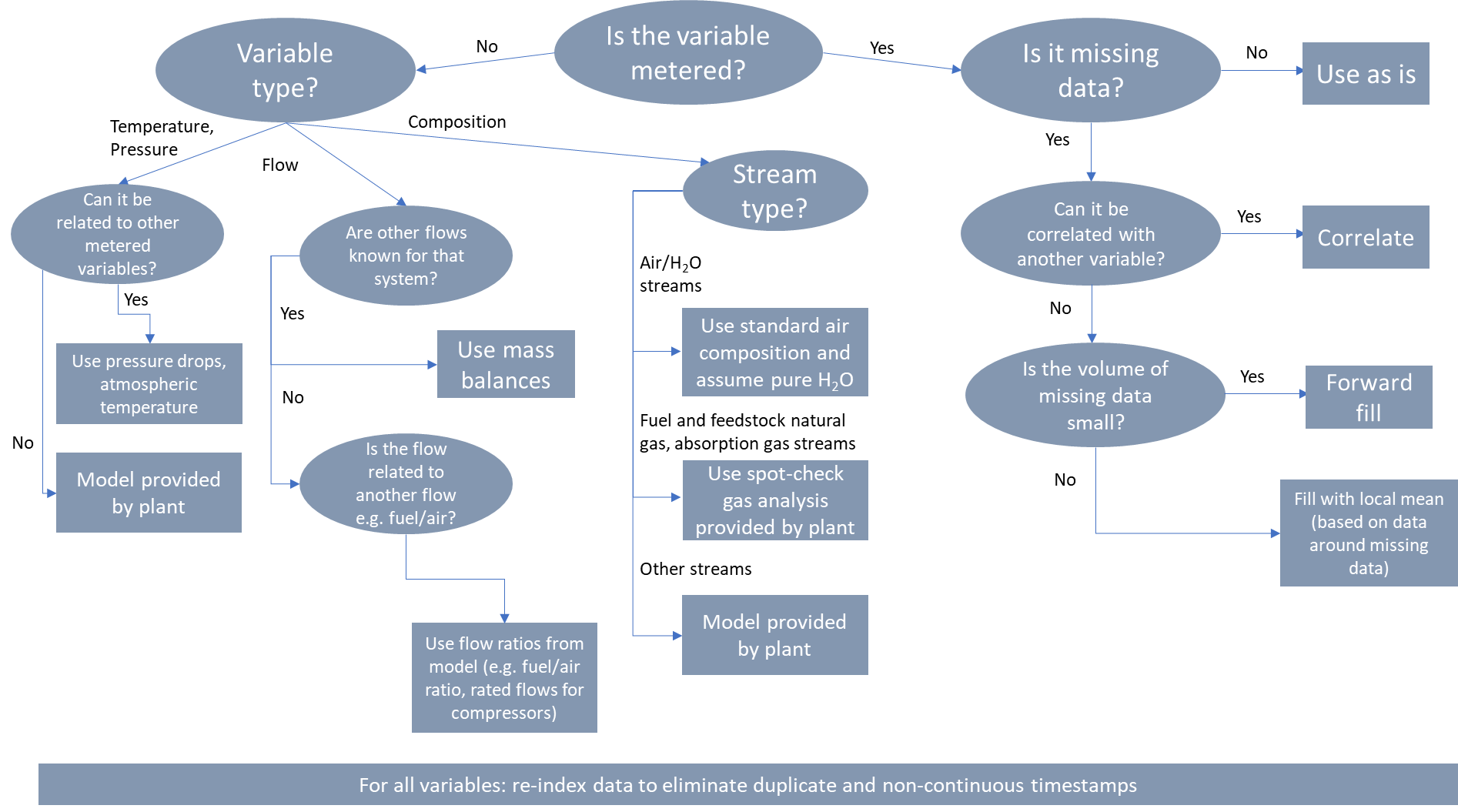


Figure 1: Strategies adopted for reconciling missing and un-metered data.

#### Missing Data – Largescale

In the case of one variable (steam output temperature) that was missing an entire year’s worth of data (2018), we needed to estimate the missing data while preserving the dynamic nature. Simple static methods include filling the missing data with the mean or an adjusted mean based on removing outliers of the existing data, using the interquartile range method (Rousseeuw & Croux, 1993):

Q1: First quartile

Q3: Third quartile

Interquartile range (IQR): Q3-Q1

Accepted values: Q1-1.5*IQR < Accepted values < Q3 + 1.5*IQR

However, to preserve the dynamic nature of the approach, we opted to find the best correlated variable with the one in question and use a simple linear regression to estimate the 2018 data. After correlating with all other 311 variables, it turns out that the steam output temperature correlates best with the steam output pressure with a Pearson correlation factor of **0.985** which is extremely high and suitable for linear regression. This regression yields an R^2^ value of **0.97.** The 2018 temperature data are then estimated based on this regression with the 2018 pressure data. While there are more sophisticated methods than this for imputing missing data, machine-learning based methods are more typically used for randomly distributed missing data rather than this case where the second year of data is missing. In addition, this issue affected only one variable so, to reduce computational complexity, we opted for a simple linear regression.

The table below indicates the effect of the steam output temperature value on the average plant efficiency, illustrating that the impact of the value choice for filling the missing data is minimal:

Table 2: Sensitivity analysis for impact of output steam temperature on overall efficiency

| **Temperature filling missing data** | **Average plant efficiency** |
| --- | --- |
| Adjusted mean (378) | 71.15% |
| Mean (359) | 71.13% |
| Correlated | 71.14% |

#### Missing Data – Small-scale

For 140 of the 311 sensors (but only 7 out of the 52 used), they were missing data for the 31^st^ of December 2017, or 0.02% of the data used. To estimate this data and avoid a gap in the analysis for the 31^st^ of December we looked at some simple methods for filling them as outlined in Figure 2b in the main text. These were:

1. Mean of the whole data series for the 2 years
2. Mean of the data for the 1 da before and after (30/12/17-1/1/18)
3. Forward filling from the last data point (30/12/17 11:59pm)

Looking at Figure 2b, we opted for a forward-filling imputation method but we performed a sensitivity analysis to investigate the effect of the method on the overall efficiency. Table 2 below indicates that it is minimal and it is worth noting this only impacts one day out of 2 years.

Table 3: Sensitivity analysis for impact of missing data imputation method on overall efficiency

| **Missing data for 31/12 imputation method** | **Plant efficiency on the 31/12** |
| --- | --- |
| **forward fill** | 71.14% |
| **Mean** | 71.30% |
| **Local mean** | 70.93% |

#### Non-unique timestamp

The timestamps of the data were corrupted in a regular way: every day at 11:54 pm the timestamp read 00:00 am instead. This was a regular issue that was easily fixable. However, there were also irregular non-unique timestamps: the most common example was a jump forward by an hour at 1am to 2am which would then repeat. This resulted in some hours being repeated. Since this was an irregular occurrence, not repeating every day or week at a specific hour, we opted for a complete re-indexing of the data from the first to last datapoint. This was done to minimise throwing out good data values only due to corrupt timestamps. None of the sensor measurements were significantly different to the data preceding and following the irregular non-unique timestamps indicating that the error was localised to the timestamp itself. In addition, the total volume of data for every variable (apart from those outlined above) corresponded exactly to the number of minutes in two years, indicating that there weren’t any missing timestamps.

#### Corrupt sensor data

As discussed in the main text, some composition sensors presented a malfunction for the period of August-September 2018. The first choice of imputation would be a correlation with another variable. Unlike the steam output temperature, no close correlation was found with the best correlation having a Pearson coefficient of 0.5 which is not good enough. We have thus opted for a local mean imputation as the next best strategy for largescale missing data. This is calculated with 1 month of data before and after the missing period to estimate a local mean.

### Data Estimation (for un-metered data)

#### Missing temperatures and pressures and flows

| **Stream** | **Temperature** | **Pressure** | **Flow** | **Source** |
| --- | --- | --- | --- | --- |
| Boiler water input |  | 44 bar |  | Model |
| Condensate input |  | 40 bar |  | Model |
| Blowdown stream | 245 ^o^C | 36.7 bar | Boiler tank input – Boiler tank output | Balancing - Model |
| Hydrogen input | 20 ^o^C |  |  | Assumption - Model |
| Absorption gas (AG) input | 30 ^o^C |  |  | Model |
| Combustion air | Atmospheric temperature^a^ | 1.01325 bar | 13.44*(Fuel gas + AG) | Balancing – Model |
| Exhaust gases |  |  | Fuel gas + AG + Combustion air | Balancing |
| Process air to secondary reformer |  | 1.55 bar pressure drop across primary reformer heaters |  | Balancing |

Table 4: Missing variables estimation assumptions

Output 1.7 MPa steam pressure and temperature are equal to input 1.7 MPa steam as they share the same pipe and are metered by the same equipment.

Output pressure from the high-temperature shift (HTS) reactor was calculated by subtracting the measured pressure drop across the reactor from the output pressure from the secondary reformer.

The combustion air input flow was calculated as a multiple of the combined fuel (fuel natural gas and absorption waste gas) mass flow. This multiple is estimated from the model ratio. The mass flow of the exhaust gas from the combustor is the sum of the mass flows of the fuel and combustion air input. One of the blowdown flows was not metered so for that tank, the blowdown flow is estimated by subtracting the metered output steam mass flow from the metered input boiler water mass flow. Finally, the final syngas product flow is also not metered so it’s estimated using a mass balance: the process inputs (steam, air, hydrogen and natural gas) are summed to yield the final product flow. The process steam input flow is not straightforward as it’s also not metered but it’s estimated from a series of mass balances around the various steam handling and raising equipment in the plant.

#### Composition

Table 5: Composition (%) for streams mostly consisting of H_2_O

| **Stream** | **1.7 MPa steam input/output** | **Blowdown flows** | **0.9 MPa steam output** | **Boiler water input** | **Condensate input** |
| --- | --- | --- | --- | --- | --- |
| **CO_2_** |  |  |  |  | 0.12 |
| **H_2_O** | 100 | 100 | 100 | 100 | 99.88 |

Table 6: Composition (%) for all other streams

| **Stream** | **Absorption gas input** | **Process/combustion air input** | **H_2_ input** | **Exhaust gases** | **Feedstock natural gas** | **Fuel natural gas** |
| --- | --- | --- | --- | --- | --- | --- |
| **CO** |  |  |  |  |  |  |
| **CO_2_** |  | 0.06 |  | 13.18 |  |  |
| **CH_4_** | 14.75 |  | 0.0269 |  | 94.7444 | 86.318 |
| **Ar** | 6.8 | 1.27 | 0.0123 | 1.25 |  |  |
| **C_2_H_6_** |  |  |  |  | 1.50475 | 1.4364 |
| **H_2_** | 12.56 |  | 17.04 |  |  |  |
| **H_2_O** |  | 1.26 |  | 12.85 |  |  |
| **N_2_** | 65.89 | 74.57 | 79.04 | 71.22 | 2.82125 | 10.4394 |
| **O_2_** |  | 22.84 |  | 1.49 |  |  |
| **C_5_H_12_** |  |  |  |  |  | 0.308 |
| **C_3_H_8_** |  |  |  |  |  | 0.480857 |

The output syngas (after the HTS, the primary and the secondary reactors) had composition measurements for CO, CO_2_ and CH_4_. Knowing the change in moles for those three substances, the extents of reactions for the following reactions were estimated:

| Methane Reforming | CH_4_ + H_2_O -> CO + 3H_2_ |
| --- | --- |
| WGS | CO + H_2_O -> CO_2_ + H_2_ |
| HC reforming | C_m_H_n_ + mH_2_O -> mCO + (m+n/2)H_2_ |

Based on these extents of reaction, the production/consumption of the rest of the substances (outside CO, CO2 and CH4) was evaluated to yield a full composition of the produced process streams from every reactor.

### Exergy Calculations

| **Substance** | **Molecular weight (kg/kmol)** | **Standard chemical exergy (kJ/kmol)** |
| --- | --- | --- |
| **CO** | 28.01 | 275,100 |
| **CO_2_** | 44.009 | 19,870 |
| **CH_4_** | 16.043 | 831,650 |
| **Ar** | 39.9 | 11,690 |
| **C_4_H_10_** | 58.12 | 2,805,800 |
| **C_2_H_6_** | 30.07 | 1,495,840 |
| **H_2_** | 2.016 | 236,090 |
| **H_2_O** | 18.015 | 9,500 |
| **N_2_** | 28.014 | 720 |
| **O_2_** | 31.999 | 3,970 |
| **C_5_H_12_** | 72.15 | 3,464,300 |
| **C_3_H_8_** | 44.1 | 2,154,000 |

Table 7: Molecular weight and molar standard chemical exergy values used in this analysis.

## Transit Exergy Efficiency

When a large amount of input exergy flows into and out of a process without participating in any transformation, then that distorts the exergy efficiency calculated using a conventional output-over-input definition, *η*. Given a constant exergy destruction value, the higher the transit exergy, the lower the ratio of exergy destruction to exergy input, therefore the larger that distortion and the difference between a conventional and transit exergy efficiency definition. The figure below aims to illustrate how a transit exergy efficiency definition, *η_tr_*, takes into account transit exergy and yields lower (and more representative) results. The results are more representative because they are calculated for the amount of exergy that actually partakes into transformations and not the entirety of the exergy. Thus, untransformed exergy such as inert gases or chemical exergy not taking part in heat transfer do not distort the efficiency value.


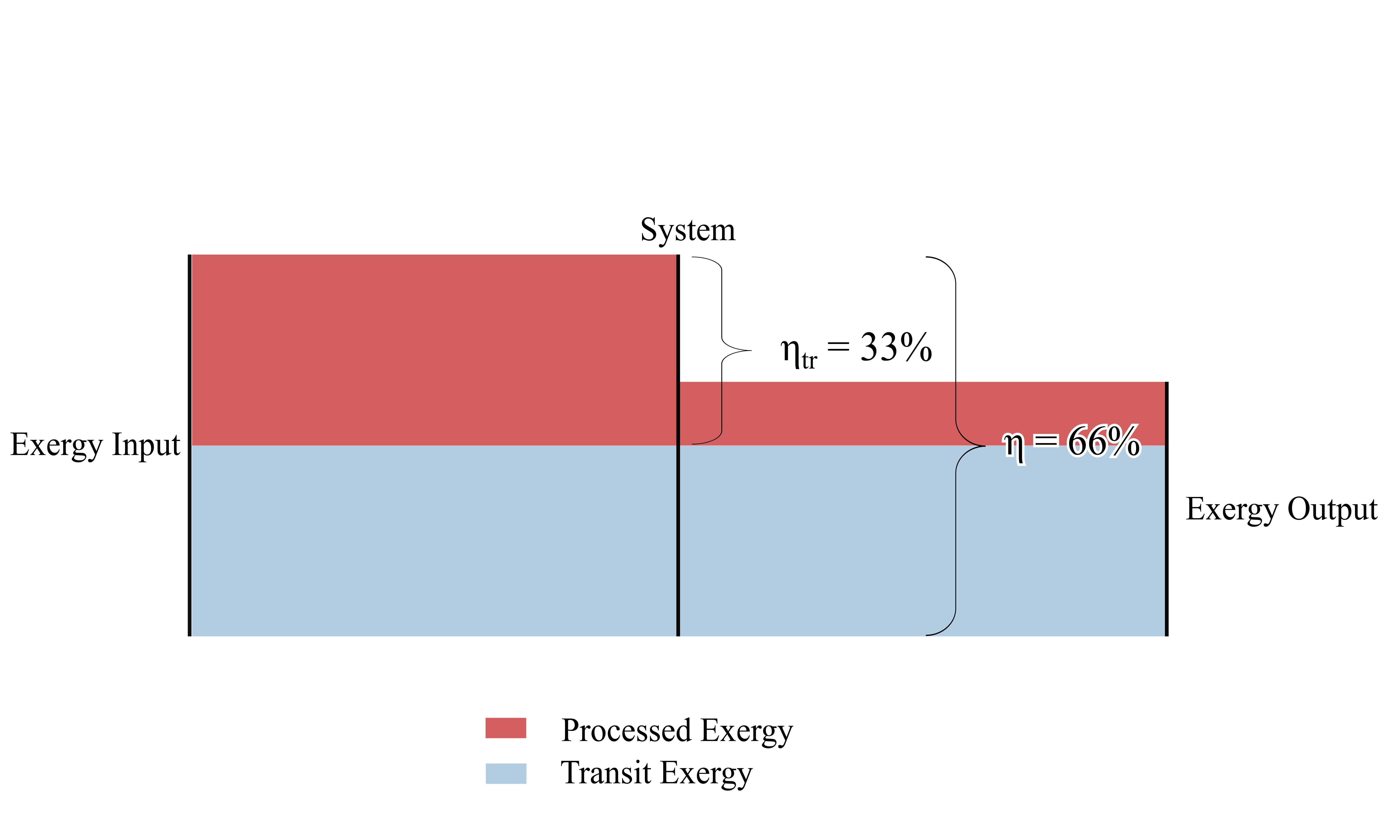


Figure 2: Illustration of difference between a conventional and transit exergy efficiency definition

## Additional references

Rousseeuw, P. J., & Croux, C. (1993). Alternatives to the median absolute deviation. *Journal of the American Statistical Association*. https://doi.org/10.1080/01621459.1993.10476408

Yaws, C. L. (2012). Yaws’ Critical Property Data for Chemical Engineers and Chemists - Table 229. Properties for Vapor/Liquid Saturation and Single Phase - Water - Knovel. Retrieved May 15, 2020, from https://app.knovel.com/web/view/itable/show.v/rcid:kpYCPDCECD/cid:kt00BKAPLF/viewerType:itble//root_slug:table-229-properties-for-vaporliquid-saturation-and-single-phase---water/url_slug:table-229-properties?q=water thermal expansion coefficient&b-q
